# Combination of absolute pitch and tone language experience enhances lexical tone perception

**DOI:** 10.1038/s41598-020-80260-x

**Published:** 2021-01-15

**Authors:** Akshay R. Maggu, Joseph C. Y. Lau, Mary M. Y. Waye, Patrick C. M. Wong

**Affiliations:** 1 Department of Linguistics and Modern Languages, The Chinese University of Hong Kong, Shatin, N.T., Hong Kong SAR China; 2 Department of Psychology and Neuroscience, Duke University, Durham, NC 27708 USA; 3 Brain and Mind Institute, The Chinese University of Hong Kong, Shatin, N.T., Hong Kong SAR China; 4 Institute for Policy Research, Northwestern University, Evanston, IL 60208 USA; 5 The Nethersole School of Nursing, Faculty of Medicine, The Chinese University of Hong Kong, Shatin, N.T., Hong Kong SAR China; 6 The Croucher Laboratory for Human Genomics, The Chinese University of Hong Kong, Shatin, N.T., Hong Kong SAR China

**Keywords:** Auditory system, Cognitive neuroscience, Language, Perception

## Abstract

Absolute pitch (AP), a unique ability to name or produce pitch without any reference, is known to be influenced by genetic and cultural factors. AP and tone language experience are both known to promote lexical tone perception. However, the effects of the combination of AP and tone language experience on lexical tone perception are currently not known. In the current study, using behavioral (Categorical Perception) and electrophysiological (Frequency Following Response) measures, we investigated the effect of the combination of AP and tone language experience on lexical tone perception. We found that the Cantonese speakers with AP outperformed the Cantonese speakers without AP on Categorical Perception and Frequency Following Responses of lexical tones, suggesting an additive effect due to the combination of AP and tone language experience. These findings suggest a role of basic sensory pre-attentive auditory processes towards pitch encoding in AP. Further, these findings imply a common mechanism underlying pitch encoding in AP and tone language perception.

## Introduction

Absolute pitch (AP), a unique ability to name or produce a given pitch without a reference^1,2^, has prevalence estimates ranging from 0.01 to 1%^2–5^. AP is known to be influenced by genetic factors^6,7^, but it is also hypothesized to be influenced by cultural factors^6^. Evidence^8–10^ suggests that AP is more commonly found in the East-Asian rather than Western populations. While evidence from the existing behavioral studies suggests that both the tone language experience^11^ and AP^8,12^ promote lexical tone perception, the effects of combination of AP and tone language experience on lexical tone perception are currently not known. In the current study, we investigated the effects of combination of AP and tone language experience on perception of lexical tones. We tested this research question behaviorally and electrophysiologically by comparing the Cantonese-speaking AP and non-AP musicians on Categorical Perception and Frequency Following Responses (FFR) of Cantonese lexical tones.

Existing evidence^9,12–16^ suggests a putative link between AP and tone language. Deutsch et al.^9^ compared 115 nontone language-speaking and 88 Mandarin-speaking students of music, on identification of piano notes. They found that Mandarin speakers outperformed the nontone-language-speakers on identification of piano notes. They also found a higher prevalence of AP in the Mandarin group. In order to rule out the potential contribution of ethnic heritage, Deutsch et al.^14^ compared musicians of Caucasian heritage with musicians of East Asian heritage (with very fluent, fluent, and nonfluent language proficiency). Overall, they found an effect of proficiency of tone language on the identification of musical notes i.e. fluent speakers of Mandarin Chinese performed better than non-fluent speakers. Further, they found that the non-fluent speakers of Chinese performed similar to the musicians of Caucasian heritage, ruling out the genetic contribution to AP. Lee and Lee^13^ tested 72 music students with AP tasks similar to Deutsch et al.^9^, but using three different timbres i.e. piano, viola, and pure tone, and they confirmed a higher prevalence of AP in Mandarin-speaking musicians. Burnham and Brooker^15^ compared nontone language speakers with and without AP on discrimination of Thai lexical tone contours presented as speech, filtered speech, and violin sounds, and they found that those with AP outperformed those without AP on identification of Thai lexical tones. Hutka and Alain^16^ investigated the combined effects of AP and tone language experience on pitch memory task using musical and non-musical stimuli by comparing 4 groups of subjects: AP+ Tone language+; AP+ Tone language–; AP– Tone Language+; and AP– Tone language–. They found that though there was an overall effect of AP on the accuracy and reaction time of the pitch memory task, there was no additive advantage of the combined AP and tone language experience on the pitch memory task. In sum, the existing evidence suggests a link between tone language experience and AP i.e. AP can be more prevalent in tone language speakers^9^, and AP promotes lexical tone perception in nontone language speakers^12^. Further, the combination of AP and tone language experience does not have an additive effect on tasks of musical pitch memory^16^. However, the effects of combination of AP and tone language experience on lexical tone perception are currently not known.

One way to examine the influence of experience-dependent effects (such as language or musical experience) on lexical tone perception is to study Categorical Perception (CP) of lexical tones^17–19^. CP is a domain-general phenomenon spanning across the disciplines of speech perception^20,21^, color perception^22^, facial expressions^23^, and music^24–27^. The hallmark of CP phenomenon is to be able to better differentiate between-categories than within-categories, even though the categories are equally-spaced physically. CP has been found to be modulated by a host of experience-dependent factors (such as language and/or music). In the context of language experience, individuals with tone language experience demonstrate an enhanced CP for lexical tones as compared to those with no tone language experience^17–19,28–30^. In other words, CP of lexical tones can be used as an indicator of proficiency of perception of lexical tones. Thus, in the current study, in order to behaviorally examine the effects of combination of AP and tone language experience on lexical tone perception, we used a CP task with lexical tone stimuli.

Another way to examine the influence of experience-dependent factors on lexical tone perception is via FFR, an electrophysiological auditory evoked response originating from the auditory brainstem and cortex^31–35^. FFR, in general, evaluates the phase locking abilities of the auditory nervous system. FFR robustly captures the pitch encoding of the stimulus, a crucial component in lexical tone perception^36–38^. Evidence suggests that FFR is influenced by long-term auditory experiences of language^39–41^, music^42,43^, and disorders^44–48^. For example, tone language speakers exhibit enhanced FFRs (i.e. enhanced magnitude, more reliable pitch) to lexical tones as compared to nontone language speakers^39,40,49^. Similarly, musicians exhibit enhanced FFRs to lexical tones as compared to non-musicians^42^. As a result, FFR has been used to examine the effects of combination of experience-dependent effects (e.g. combined language and music experience^36^).

**Figure 1:**
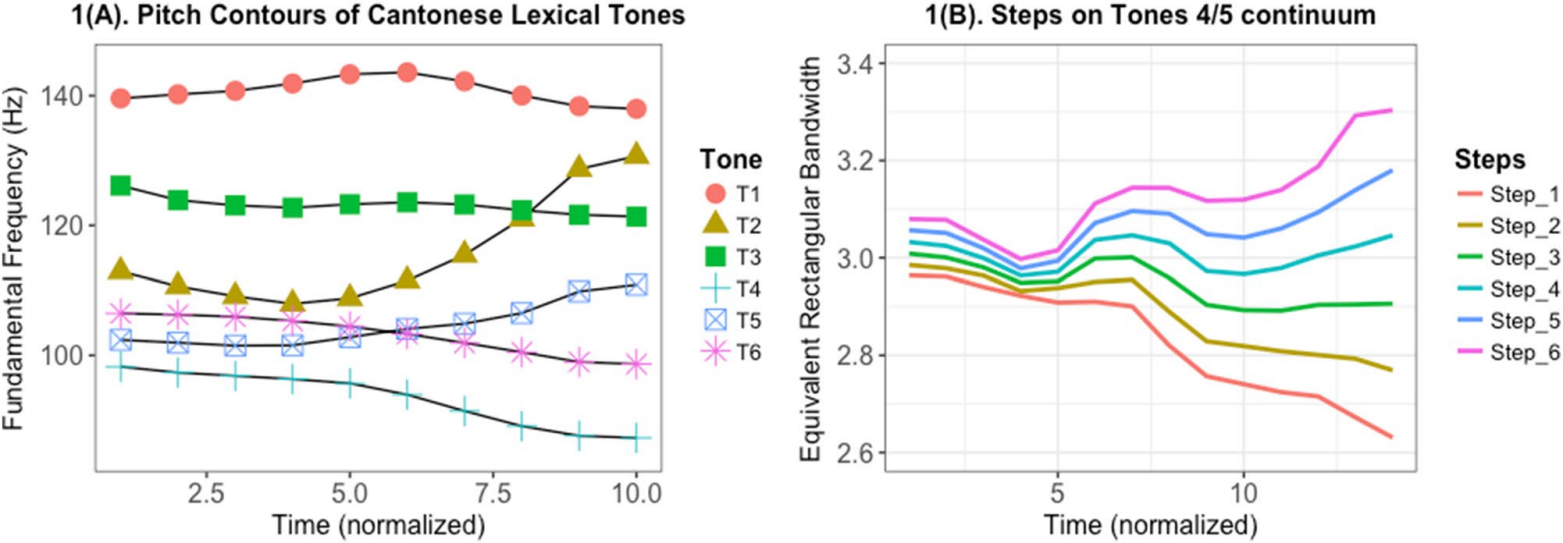
(**A**) Pitch contours of lexical tones in Cantonese. F0 ranges: T1: 135–146 Hz, T2: 105–134 Hz, T3: 120–124 Hz, T4: 85–99 Hz, T5: 102–113 Hz, T6: 100–106 Hz; (**B**) Pitch contours of the six-step T4/T5 equidistant continuum. Step 1 = original T5; Step 6 = original T4.

In the current study, we investigated the effects of combination of AP and tone language experience on lexical tone perception. We investigated this behaviorally (via Categorical Perception) and electrophysiologically (via FFR) using Cantonese lexical tone stimuli. Cantonese, a Chinese language spoken mostly in Hong Kong and southern parts of China, has six lexical tones (Fig. 1A) consisting of three level tones (Tone 1: high, Tone 3: mid, Tone 6: low), two rising tones (Tone 2: high-rising, Tone 5: low-rising), and a falling (Tone 4) tone. For our CP experiment (Experiment 1), we used a 6-step F0 continuum between Tone 4 (falling) and Tone 5 (low-rising) (Fig. 1B). For our FFR experiment (Experiment 2), we used all six lexical tones of Cantonese. In the current study, we compared Cantonese musicians with and without AP on the lexical tone CP task and FFR. If the effect of combined AP and tone language experience was additive, Cantonese musicians with AP would outperform those without AP on both CP and FFR experiments. Conversely, if the combined effect of AP and tone language was not additive, there would be no difference between those with and without AP on CP and FFR experiments. More specifically, we predicted a sharper identification curve for the AP group in the CP identification task and a group (AP vs. Non-AP) $\times$ category (across vs. within) interaction in the CP discrimination task. For the FFR experiment, we predicted that the AP group will outperform the Non-AP group on the FFR metrics: (1) Stimulus-to-response correlation; (2) Pitch Error; (3) Signal-to-Noise Ratio; and (4) Root mean square amplitude.

## Results

**Figure 2:**
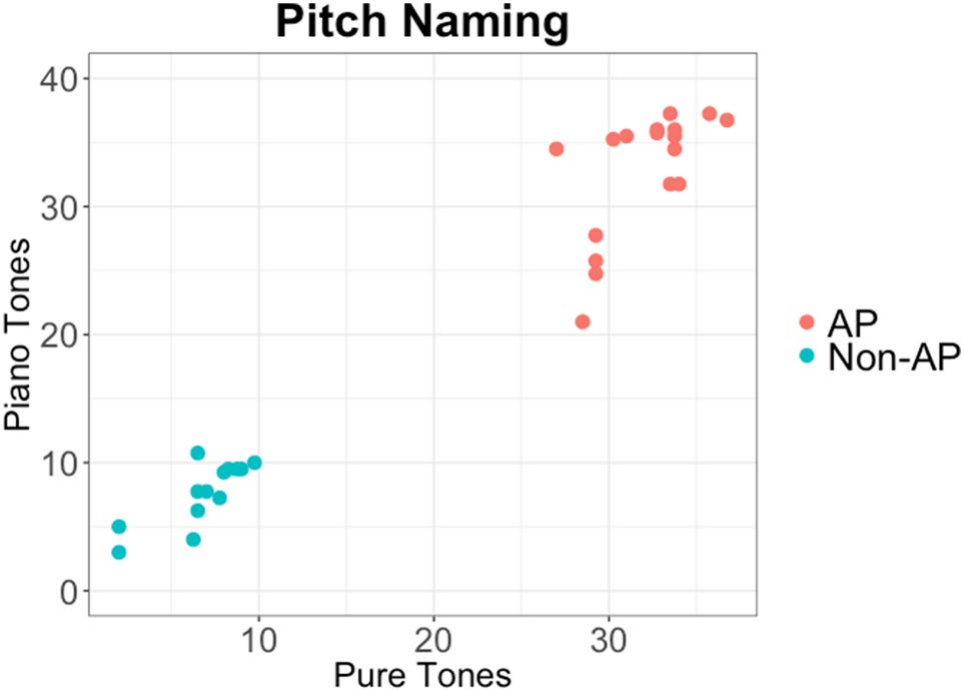
Distribution of AP and Non-AP subjects across the identification of piano (Y-axis) and pure tones (X-axis).

In the current study, we recruited 30 subjects (17 AP and 13 Non-AP). Subjects were identified into the respective groups (AP and Non-AP) based on their scores on the pitch naming task^6^ (Fig. 2). The subjects in the two groups were similar in terms of age, musical experience, and IQ.

## Experiment 1: Categorical perception

A CP task was created with a 6-step pitch contour continuum between Cantonese lexical Tones 4 and 5 (Fig. 1B). Subjects were then tested on identification and discrimination between categories.

### Identification task

**Figure 3:**
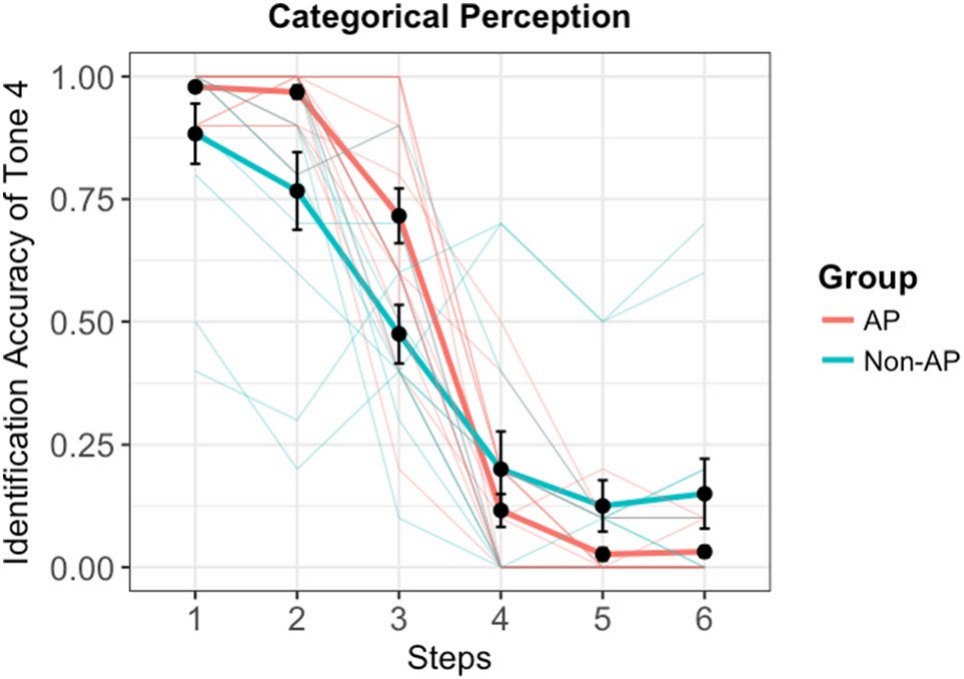
Comparison of AP and Non-AP groups on the identification accuracy of steps as Tone 4. Light colored lines depict individual subject curves. Error bars =  ± 1 SEM.

Subjects were presented stimuli from the six steps and they were asked to label the heard stimulus as Tone 4 or 5 in a Two-Alternative Forced Choice task. Figure 3 reveals a comparison between the AP and Non-AP group on the identification accuracy for the stimuli steps as Tone 4. Overall, AP group exhibited a sharper identification curve as compared to the Non-AP group. Participants’ identification response for each stimulus as T4 or T5 was fitted into a multiple logistic regression model, with group (AP and Non-AP) and step (step 1–6 of the continuum) as well as the group × step interaction as predictors.

The logistic regression model was significant (χ2 = 1034.6, *p* < 0.001), with both step (β = 2.17067, Z = 5.955, *p* < 0.001) and group (β = 1.46028, Z = 18.258, *p* < 0.001) as significant predictors. Crucially, the step x group interaction was significant (β = − 0.57334, Z = − 5.538, *p* < 0.001), suggesting that the group status was a predictor in how the continuum was categorized into the two lexical tone categories. To break down the interaction, identification responses from each group were further analyzed using two separate logistic regressions with step as a predictor. Sharpness of the categorical boundary for each group was calculated as the β value of step (b1) in each group-level logistic regression model, and the mean categorical boundary (in 1–6 steps) for each group was calculated as − b0/b1, where b0 us the β value of the intercept of the fitted logistic curve^19^. Group level results are presented in Table 1. Results suggest that the AP group had a later mean categorical boundary (3.332991) than the Non-AP group (3.04161). The b1 of the AP group (1.46028) was also higher than the Non-AP group (0.88694). These results suggest that categorical perception between T4/T5 was sharper for the AP group relative to the Non-AP group, which also drove the significant group × step interaction in the multiple logistic regression model. However, it should be noted that the difference in the extreme ends of the continuum were contributed by two subjects in the non-AP group who performed around chance (see Fig. 3).

**Table 1:** Estimates of regression coefficients (b0, b1) and the derived mean categorical boundary (− b0/b1) for each group (AP and non-AP).

Group	b0	b1	− b0/b1
AP	− 4.86710	1.46028	3.332991
Non-AP	− 2.69644	0.88694	3.04161

### Discrimination task

**Figure 4:**
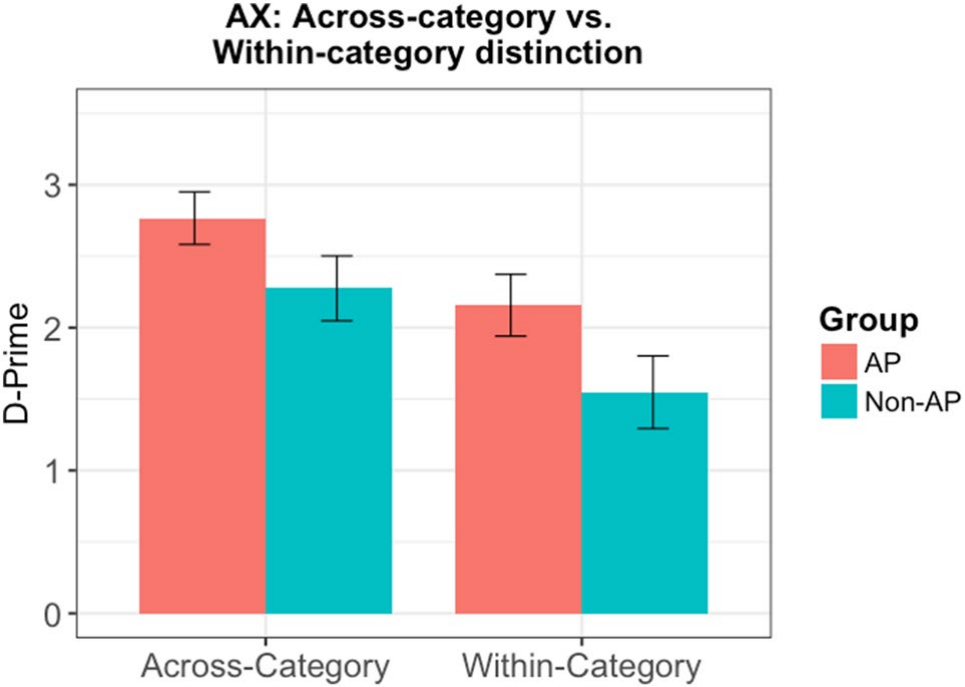
Mean d’ score for across- and within-category distinctions for AP and Non-AP groups. Error bars =  ± 1 SEM.

This experiment aimed at furthering our understanding towards categorical perception in AP versus Non-AP subjects. In this experiment, AP and Non-AP subjects discriminated an across-category distinction, and a within-category distinction along the T4/T5 continuum in an AX discrimination paradigm. Participants d’ scores for the discrimination of across- and within-category distinctions were first calculated by subtracting the Z-score of the False Alarm rate from the Z-score of the Hit rate. Figure 4 shows the mean d’ score for across- and within-category distinctions for AP and Non-AP groups.

The d’ scores were analyzed using a 2 $\times \hspace{0.17em}$2 mixed ANOVA with group (AP vs. Non-AP) and acrosswithin (Across- vs. within-category distinctions) as factors. The ANOVA revealed a main effect of acrosswithin [F(1,28) = 15.743, *p* < 0.001], confirming that the T4/T5 continuum was perceived categorically. Interestingly, there was a marginal effect of group [F(1,28) = 4.196, *p* = 0.05] suggesting that the AP subjects are more sensitive to both across- and within-category distinctions. However, the group $\times \hspace{0.17em}$acrosswithin interaction was not significant [F(1,28) = 0.121, *p* = 0.73].

## Experiment 2: FFR

**Figure 5:**
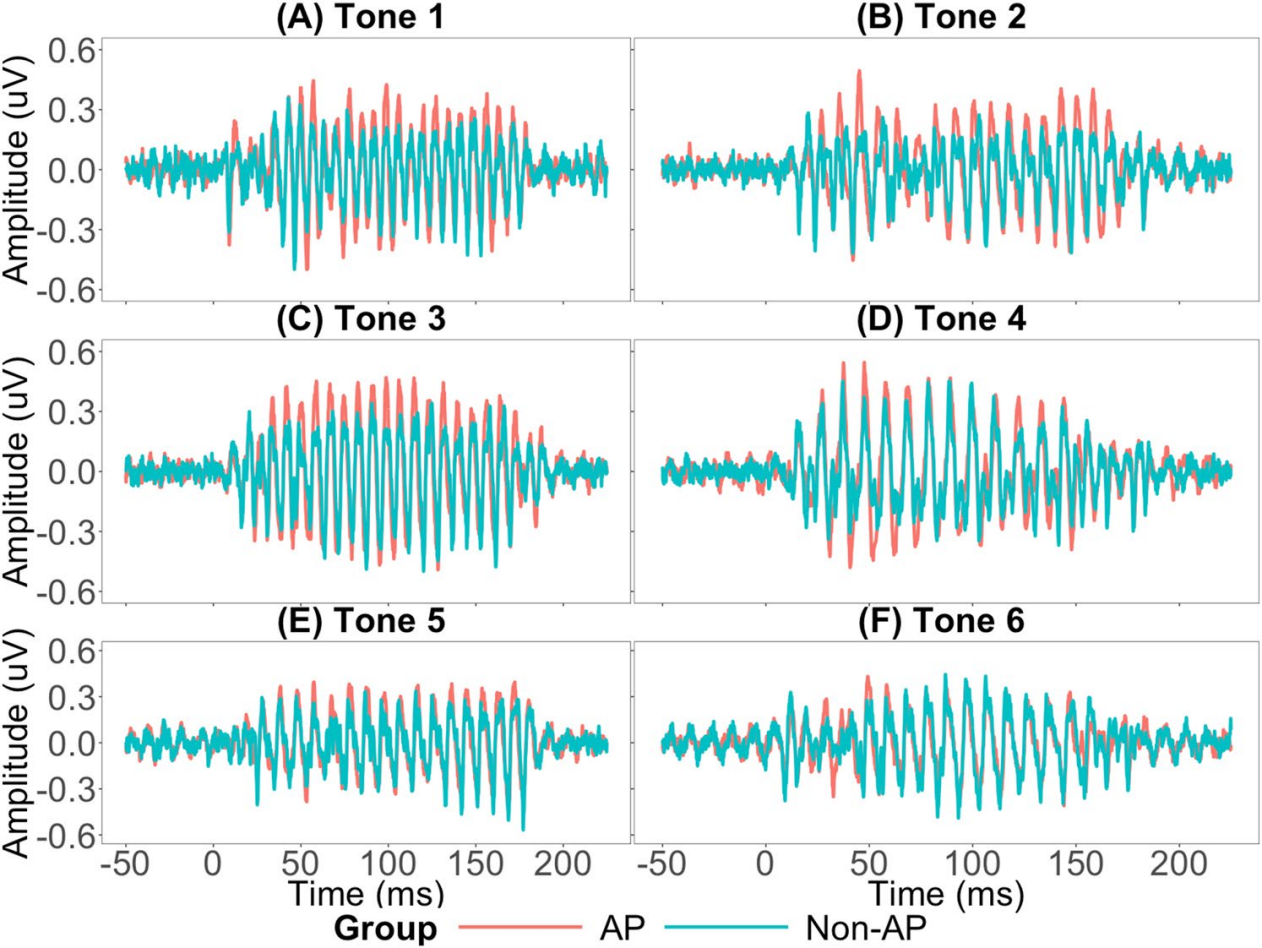
Grand-averaged FFR waveforms of AP and non-AP group for lexical tones 1–6 (**A**–**F**). Overall, the AP group exhibited greater FFR amplitude as compared to the Non-AP group.

Figure 5 shows the grand-averaged FFR waveforms of the AP and Non-AP groups for the six Cantonese lexical tones. Separate 2 (Group: AP vs Non-AP) × 6 (Tones: 6 lexical tones) ANCOVAs were conducted for each of the FFR measures, namely, stimulus-to-response correlation, pitch error, signal-to-noise ratio, and root-mean-square amplitude, with age as a covariate.

**Figure 6:**
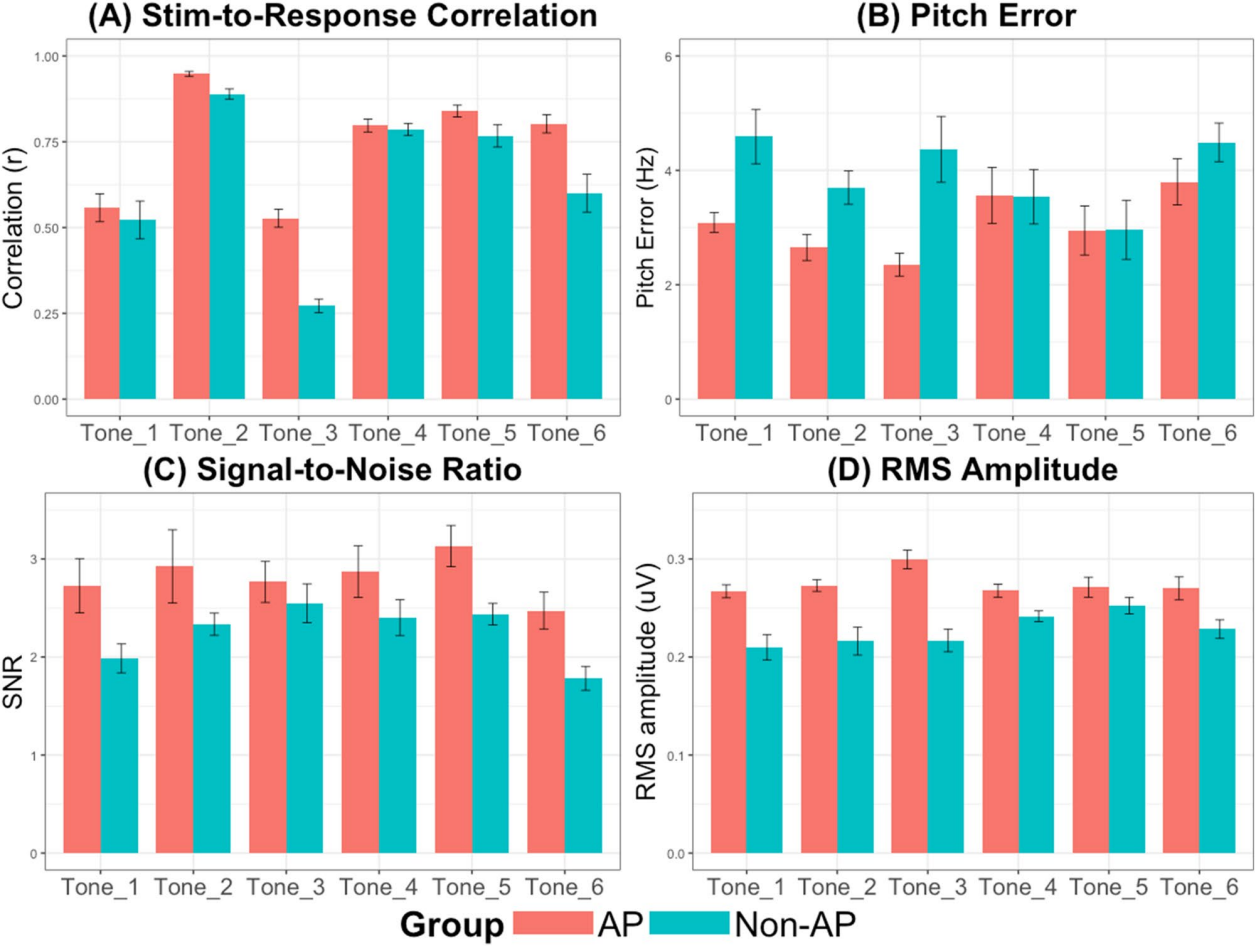
Comparison of AP and Non-AP group across (**A**) Stimulus-to-Response correlation; (**B**) Pitch Error; (**C**) Signal-to-Noise Ratio; and (**D**) RMS amplitude. Overall, the AP group outperformed the Non-AP group on all the measures. Error bars =  ± 1 SEM.

- Stimulus-to-Response Correlation: There was a main effect of group, F(1,27) = 30.93, *p* = 0.00, ${\eta }_{p}^{2}$ = 0.53, main effect of tone, F(5,135) = 33.98, *p* = 0.000, ${\eta }_{p}^{2}$ = 0.68, but no significant group $\times$ tone interaction, F(5,135) = 2.87, *p* = 0.101, ${\eta }_{p}^{2}$ = 0.09. There was no significant effect of age, F(1,27) = 1.78, *p* = 0.193, ${\eta }_{p}^{2}$ = 0.06. Stimulus-to-response correlation for Tone 2 was the greatest and for Tone 3 it was the lowest. The main effect of group was mostly driven by difference between the groups on Tones 2, 3, and 6 (Fig. 6A). Post-hoc paired t-test revealed that Tone 2 significantly differed from all the other tones (Tone 2–Tone 1: t(29) = − 11.87, *p* = 0.000; Tone 2–Tone 3: t(29) = 19.33, *p* = 0.000; Tone 2–Tone 4: t(29) = 8.82, *p* = 0.000; Tone 2–Tone 5: t(29) = 6.26, *p* = 0.000; Tone 2–Tone 6: t(29) = 6.8, *p* = 0.000).
- Pitch Error: There was a main effect of group, F(1,27) = 7.69, *p* = 0.01, ${\eta }_{p}^{2}$ = 0.22, no main effect of tone, F(5,135) = 1.49, *p* = 0.197, ${\eta }_{p}^{2}$ = 0.05, and no significant group $\times \hspace{0.17em}$tone interaction, F(5,135) = 1.17, *p* = 0.153, ${\eta }_{p}^{2}$ = 0.06. There was no significant effect of age, F(1,27) = 0.02, *p* = 0.895, ${\eta }_{p}^{2}$ = 0.001. The AP group showed lower pitch error than the Non-AP group on Tones 1, 2, and 3 (Fig. 6B).
- Signal-to-Noise ratio (SNR): There was a main effect of group, F(1,27) = 7.62, p = 0.014, ${\eta }_{p}^{2}$ = 0.22, no main effect of tone, F(5,135) = 0.76, p = 0.581, ${\eta }_{p}^{2}$ = 0.03, and no significant group $\times \hspace{0.17em}$tone interaction, F(5,135) = 0.41, *p* = 0.834, ${\eta }_{p}^{2}$ = 0.15. There was no significant effect of age, F(1,27) = 0.39, *p* = 0.538, ${\eta }_{p}^{2}$ = 0.01. AP and Non-AP groups differed the least on Tones 3 and 4 (Fig. 6C).
- Root-mean-square (RMS) amplitude: There was a main effect of group, F(1,27) = 24.93, *p* = 0.00, ${\eta }_{p}^{2}$ = 0.48, no main effect of tone, F(5, 135) = 0.83, *p* = 0.532, ${\eta }_{p}^{2}$ = 0.03, and a significant group × tone interaction, F(5,135) = 4.7, *p* = 0.001, ${\eta }_{p}^{2}$ = 0.15. There was no effect of age, F(1,27) = 0.57, *p* = 0.458. AP and Non-AP groups differed the most on Tones 1, 2, and 3 (Fig. 6D).

Further, even after normalizing the age by musical experience (i.e. age/musical experience), we found no significant effect on any of the above measures (Stimulus to response correlation: F(1,27) = 0.827, *p* = 0.371; Pitch Error: F(1,27) = 0.316, *p* = 0.579; SNR: F(1,27) = 0.03, *p* = 0.875; RMS amplitude: F(1,27) = 2.04, *p* = 0.164).

## Discussion

In the current study, we investigated the effects of combination of AP and tone language experience on lexical tone perception, via behavioral and electrophysiological measures. Our main finding from the current study is that the combination of AP and tone language experience outweighs the effect of tone language experience alone, in promoting lexical tone perception. These findings have implications towards the possibility of common neural mechanisms underlying pitch encoding in AP and tone language speakers.

Previous findings suggest that the experience-dependent effects of language and music influence lexical tone perception^36,41,42,50,51^. For example, musicians exhibit enhanced CP of lexical tones^52^, and enhanced neural encoding of lexical tones^42^, as compared to non-musicians. Similarly, tone language experience has been found to promote lexical tone perception^40^. For example, Mandarin speakers exhibit enhanced lexical tone perception as compared to English speakers. However, a combination of the experience-dependent effects of language and music has been found to have no additive effect on lexical tone perception but an enhanced musical pitch perception^36^. For example, Cantonese musicians demonstrate similar neural encoding of lexical tones, but an enhanced neural encoding of musical tones, as compared to Cantonese non-musicians. These findings suggest differential mechanisms underlying the musical and linguistic pitch encoding in tone language musicians. In comparison, the findings from the current study demonstrate additivity of effects of tone language experience and AP on lexical tone perception, reinforcing the view that AP and tone language may have a common perceptual substrate^8,15,53^.

Since FFR originates from the cortex^31,32^ and the brainstem^33,43,54,55^, the current findings are consistent with the previous findings on the contribution of cortical processes towards pitch processing in AP^56–60^. More specifically, the current findings are in line with the role of basic sensory processes towards pitch processing of AP in the auditory cortex. For example, McKetton et al.^60^ compared 20 AP musicians, 20 non-AP musicians, and 20 control musicians on processing of ascending and descending frequency sweeps during fMRI testing to map out frequency tuning characteristics in the primary auditory cortex, the rostral part of auditory cortex, and the rostro-temporal parts of auditory cortex. They not only found bilaterally larger Heschl’s gyri in AP musicians than in the other two groups, but also possible contributions from a range of neurons in the different areas of the Heschl’s gyrus towards pitch encoding. In the current study, since the F0 of (all but one of) our stimuli was > 100 Hz, the elicited FFRs might have also been influenced by the processes in the brainstem^33,35,54^. The possibility of contribution of brainstem processes towards pitch in AP finds support from the model proposed by Ross et al.^62^. According to this model, pitch processing in AP could be a result of enhanced synaptic activity at the level of the inner hair cells and auditory nerve. As the inferior colliculi (in the auditory brainstem) is considered to be a site for the reintegration of spectral information^61^ arriving from the lower structures in the auditory neural pathway^62^, we speculate that the FFRs could be influenced by this information. In sum, the enhanced FFR pitch encoding in AP subjects, in the current study, could be a result of contributions from both the auditory brainstem and cortex.

Evidence from the previous cortical electrophysiological studies examining the role of pre-attentive auditory processes in AP processing has largely been mixed^58,59^. The current findings reveal the contributions of pre-attentive auditory processes towards pitch processing in AP. One of the key reasons why this study, unlike the previous studies, was able to find the effect of pre-attentive auditory processes, was probably due to the type of auditory evoked potential (AEP) used. In order to evaluate the pitch processing abilities in AP versus non-AP subjects, previous studies mainly relied on mismatch negativity (MMN) and/or P3a, which are AEPs obtained via subtracting the waveforms generated by deviant from standard pure-tone stimuli in an oddball paradigm. These AEPs have been suggested as being indices of detection of change rather than being pitch-specific^63^, with the result that they can be affected by other factors, such as the ability to detect a change. In comparison, FFRs are pitch-specific AEPs, and in general, are more robust and reliable than MMN. Thus, the current study offers a technical advantage as compared to the previous electrophysiological studies in this area of research.

At this juncture, we suggest that the current findings should be construed in the context of tone languages. There is a growing need for future studies in nontone languages to examine the contribution of neural pitch encoding in AP using a combination of cortical and subcortical AEPs. To conclude, our study demonstrated that a combination of AP and tone language experience enhances neural encoding of lexical tones as compared to tone language experience alone. In order to further understand the levels of neural encoding of pitch in AP and the interplay between the subcortical and cortical levels of pitch in AP, future studies could consider conducting simultaneous recordings of pitch-specific subcortical and cortical evoked potentials^63^. Furthermore, the current findings exhibit a difference between AP and non-AP at a group level. Our AP group falls in the AP1 category^6^. Future studies need to be conducted to understand the neural encoding in other subtypes of AP.

## Methods

### Participants

**Figure 7:**
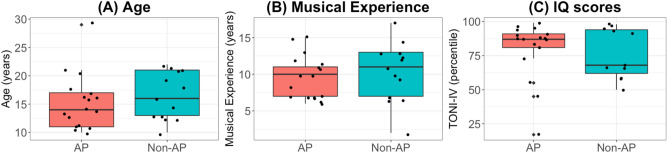
Comparison of AP and Non-AP group across (**A**) Age; (**B**) musical experience; and (**C**) IQ scores. The groups did not differ on any of these measures.

We recruited 17 subjects (mean age = 15.29, SD = 4.87) in the AP group and 13 subjects (mean age = 16.3, SD = 4.21) in the non-AP group. Their peripheral hearing sensitivity was within 25 dB HL on 500 Hz, 1000 Hz, 2000 Hz and 4000 Hz. They had no history of middle ear pathology, no speech-language dysfunction and no known anatomical or neurological defects. The subject groups were similar in terms of age, musical experience and IQ (TONI-IV) (Fig. 7). Further, the groups were also matched on the start age of musical instruction (AP: mean = 4.44 y, SD = 1.12; Non-AP: mean = 4.84 y, SD = 1.34). The study was approved by and conducted in accordance with the guidelines and regulations of the Joint Chinese University of Hong Kong—New Territories East Cluster Clinical Research Ethics Committee. Informed consent was obtained from each participant.

### Pitch naming test

Stimuli for pitch naming included 40 pure tones and 40 piano tones obtained from Baharloo et al.^6^. The test for pitch naming was adapted from Baharloo et al.^6^ and coded on E-prime 2.0 professional. Testing for pure tone identification and piano tone identification was conducted separately and counterbalanced across subjects. Each test consisted of 4 blocks with 10 trials in each block with 3-s intervals between each trial. On the keyboard, the keys were labeled as musical notes. The output from these tests was routed via headphones to the subjects. The subjects’ task was to hear the stimuli and press the relevant button on the keyboard that corresponded to the note of the pitch they heard. No feedback was provided during the testing. Each correctly-identified trial was awarded one point and if the subjects mis-identified a trial by a semitone, they were awarded 3/4 of a point^1,6,10^. We included the subjects that belonged to AP1 category (i.e., pure tone score > 24.49) and those who belonged to Non-AP category (i.e., pure tone and piano score > 3.25 but < 11.25). Figure 2 reveals the distribution of the subjects.

### Stimuli

Stimuli in the current study consisted of Cantonese lexical tones. Lexical tones superimposed on the syllable /ji/ were recorded, making six unique words: /ji1/ “doctor,” /ji2/ “chair,” /ji3/ “meaning,” /ji4/ “son,” /ji5/ “ear” and /ji6/ “justice”. Figure 1A shows the pitch contours of the six Cantonese lexical tones (F0 ranges: T1: 135–146 Hz, T2: 105–134 Hz, T3: 120–124 Hz, T4: 85–99 Hz, T5: 102–113 Hz, T6: 100–106 Hz)^36,37,64^. The stimuli were produced by a phonetically-trained 25-year old male native speaker of Cantonese, recorded using a Shure SM10A microphone and Praat^65^ at a sampling rate of 44,100 Hz. Five versions of the stimuli with durations normalized to 150, 175, 200, 225, and 250 ms were created. After a speech identification task taken by 12 native Cantonese speakers, 175 ms were decided as the stimuli for the experiment as they were most consistently and correctly identified by the native speakers. All six lexical tones (Fig. 1A) were used for the FFR experiment (Experiment 2). Stimuli for the CP experiment (Experiment 1) consisted of a continuum of F0 contour constructed with Tones 4 and 5 as the endpoints. In order to construct a continuum, the F0 contours of T4 and T5 were estimated by Praat^65^ followed by computing the F0 values across 14 points of the pitch contours of Tones 4 and 5 using an auto-correlation-based method. For each adjacent step along the continuum, the two F0 contours (across 14 time points) had an averaged Euclidean distance of 0.06 ERB. The F0 contours of the six stimuli are presented in Fig. 1B. Each F0 contour was then superimposed on the /ji/ syllable of the normalized T5 stimulus using the built-in overlap-add synthesis method in Praat, resulting in six tokens of auditory stimulus.

### Procedure

#### Experiment 1: CP of lexical tones

##### Identification

In the experiment, both groups of participants identified each of the six steps of the continuum as an instance of T4 or T5 in a two-alternative force choice identification paradigm. The experiment was programmed and presented with E-Prime 2.0 professional using a Dell Optiplex 9010 desktop computer connected to a Dell U2312HM LCD monitor and Sennheiser 280 HD Pro headphones. The participant was verbally instructed prior to the experiment to identify the stimuli as /ji4/ `son’ or /ji5/ `ear’ with two designated keys on the keyboard. Ten repetitions of the six steps of the continuum were presented, resulting in a total of 60 trials for each participant. Presentation order of the 60 trials was randomized. Each trial began with a fixation cross appearing at the center of the screen. After 500 ms, the auditory stimulus token was presented through the headphones. A screen indicating the designated keys (1 for /ji4/, 2 for /ji5/) and the two corresponding words in Chinese characters (兒 and 耳, respectively) appeared simultaneously with the auditory stimulus presentation. The participant was instructed to respond using the designated keys on the numpad of the keyboard. The next trial was presented when a response was recorded.

##### Discrimination

This part of the experiment aimed at furthering our understanding towards categorical perception in AP versus Non-AP subjects. In this experiment, AP and Non-AP subjects discriminated an across-category distinction, and a within-category distinction along the T4/T5 continuum in an AX discrimination paradigm.

Visual inspection of the identification curves by adult native Cantonese speakers suggest that the categorical boundary between the T4/T5 continuum is around step 3. The visually-determined categorical boundary is confirmed by Experiment 2a, which found that the mean categorical boundary for both groups is also around 3 (3.332991 and 3.04161 respectively). As a result, step 2/step 4 of the continuum was used as an across-category distinction, while the step 4/step 6 was used as a within-category distinction for discrimination.

Participants discriminated the across- (step 2/step 4) and within-category (step 4/step 6) distinctions in an AX discrimination paradigm. In each trial of the AX discrimination paradigm, participants were presented with two sounds, whereby they had to judge whether the two sounds were same or different. The experiment was programmed and presented with E-Prime 2.0 professional using an identical computer as in the previous task. The participants were verbally instructed prior to the experiment to identify whether the two sounds they hard were the same or different by pressing two designated keys on the keyboard. 32 trials were randomly presented to each participant. The 32 trials consisted of 4 repetitions of same (AA and BB trials) and different (AB and BA trials) trials for both across- (step 2/step 4) and within-category distinctions. Each trial began with a fixation cross appearing at the center of the screen. After 500 ms, the first auditory stimulus token was presented through the headphones. The second auditory stimulus token was presented with an inter-stimulus-interval of 425 ms. A screen indicating the designated keys (1 as same, 2 as different) along with the two corresponding choices in Chinese characters (相同 `same’ and 不同 `different’) appeared simultaneously with the presentation of the second auditory stimulus. The participant was allowed 3 s to respond, or else the next trial was presented.

#### Experiment 2: FFR of lexical tones

*Stimuli presentation* 3000 trials of each stimulus were presented using AudioCPT module of STIM2 Neuroscan (Compumedics, El Paso, TX) with an interstimulus interval which jittered between 74 and 104 ms in alternating polarity^36–38,66,67^ routed binaurally via Compumedics 10 Ω insert earphones. The order of stimuli presentation was counterbalanced across participants. Participants were asked to relax and ignore the stimuli.

*Data acquisition and pre-processing* Electrophysiological data were acquired using a 4 electrode montage Cz (active)-(M1 + M2)-lower forehead (ground) using a Neuroscan Synamps amplifier connected to Curry Neuroscan 7.05 (Compumedics, El Paso, TX). Inter-electrode impedances were maintained at or below 1 kΩ. Data were collected at a sampling rate of 20,000 Hz. Data pre-processing, consisting of baseline correction (− 50 ms), artifact rejection (± 35 µV), band-pass filtering (80–5000 Hz), epoching (− 50 to 275 ms) and averaging, was conducted using Curry Neuroscan 7.05 (Compumedics, El Paso, TX) system for analysis. None of the FFR recordings had more than 10% rejections (i.e., < 300 rejections).

*Data analysis* The FFR data were further band-pass filtered in the range of 80–2500 Hz to eliminate high frequency components of EEG noise^51,67^ and slower cortical components from the subcortical data. Further, the data were transformed from the temporal to the spectral domain using a short term Fourier transform with a 40-ms sliding window in 1-ms steps^68^. Spectral peaks extracted during the FFT procedure were connected to obtain the pitch contours of the FFRs. From the FFR pitch information, the following measures^36–38,67,68^ were extracted to compare the subjects with and without absolute pitch: (a) *Stimulus-to-response Correlation (ranging from *− 1* to* + 1) which is a simple correlation between the pitch contour of the FFR and that of the stimulus. Higher positive stimulus-to-response correlation reflects a better recapitulation of the stimulus at the subcortical level; (b) *Signal-to-Noise Ratio (SNR)* which refers to the ratio of RMS amplitude of the signal to that of the pre-stimulus period (-50 ms); (c) *Pitch Error (in Hz)* which refers to the average Euclidean distance between the stimulus pitch contour and the response pitch contour. The lower the pitch error, better the subcortical pitch encoding; (d) *Root Mean Square (RMS; in µV)* amplitude which is the magnitude of FFR signal from the onset. Higher magnitude refers to better pitch representation at the subcortical level.
